# UK news media representations of smoking, smoking policies and tobacco bans in prisons

**DOI:** 10.1136/tobaccocontrol-2017-053868

**Published:** 2018-02-19

**Authors:** Amy Robinson, Helen Sweeting, Kate Hunt

**Affiliations:** MRC/CSO Social and Public Health Sciences Unit, University of Glasgow, Glasgow, UK

**Keywords:** cessation, priority/special populations, public policy, secondhand smoke, media

## Abstract

**Background:**

Prisoner smoking rates remain high, resulting in secondhand smoke exposures for prison staff and non-smoker prisoners. Several jurisdictions have introduced prison smoking bans with little evidence of resulting disorder. Successful implementation of such bans requires staff support. As news media representations of health and other issues shape public views and as prison smoking bans are being introduced in the UK, we conducted content analysis of UK news media to explore representations of smoking in prisons and smoke-free prisons.

**Methods:**

We searched 64 national and local newspapers and 5 broadcast media published over 17 months during 2015–2016, and conducted thematic analysis of relevant coverage in 106 articles/broadcasts.

**Results:**

Coverage was relatively infrequent and lacked in-depth engagement with the issues. It tended to reinforce a negative view of prisoners, avoid explicit concern for prisoner or prison staff health and largely ignore the health gains of smoke-free policies. Most coverage failed to discuss appropriate responses or support for cessation in the prison context, or factors associated with high prisoner smoking rates. Half the articles/broadcasts included coverage suggesting smoke-free prisons might lead to unrest or instability.

**Conclusions:**

Negative news media representations of prisoners and prison smoking bans may impact key stakeholders’ views (eg, prison staff, policy-makers) on the introduction of smoke-free prison policies. Policy-makers’ communications when engaging in discussion around smoke-free prison policies should draw on the generally smooth transitions to smoke-free prisons to date, and on evidence on health benefits of smoke-free environments and smoking cessation.

## Introduction

Smoking rates among prisoners remain very high (two to four times general population rates in all studies internationally),^1 2^ reflecting characteristics of both prisoner populations and prison environments. Prisoners are disproportionately from more disadvantaged communities and have high rates of mental health problems and substance abuse. These characteristics are associated with both smoking and resistance to smoking cessation^1–3^

In countries without prison smoking bans, smoking is a social norm within prisons.^2–4^ Smoking is described by prisoners as a way of dealing with boredom and isolation, and perceived, by prisoners and staff, to help prisoners cope with stress.^1–4^ Prisons present a challenging setting for cessation services: smoking cessation may have lower priority than other (health) issues and is complicated by the transient nature of many prison stays and potentially low motivation to quit.^1 2^

There is evidence of direct links between prisoner smoking and smoking-related cancers^5^ and all-cause cancers,^6^ but those exposed to secondhand smoke (SHS) also experience health impacts.^7^ High SHS levels within prisons have been reported in the USA,^8 9^ Ireland,^10^ England and Wales^11 12^ and Scotland.^13^

Concerns around prisoner and staff health, legal challenges from non-smokers, and safety, maintenance and insurance costs^2 14^ have prompted several jurisdictions to introduce partial or total prison smoking bans. These have been associated with reduced prisoner smoking^15^ and SHS,^8 9 16^ and positive impacts on prisoner health.^17 18^ Pre-ban anxieties about disorder have generally proved unfounded,^2 19 20^ although increases in tobacco black markets^14 21^ and riskier smoking practices^22^ have been reported.

Evidence suggests the success of any ban depends on prison staff support, careful preparation and communication with prisoners and staff, and provision of cessation support.^1 2 20^ A key factor shaping public and policy-makers’ understandings and opinions is the news media.^23 24^ However, media news stories are not simply (all the) ‘facts’. Their content has been described as “a particular version of reality” (Rooke and Amos, p508)^5^, influenced by issues such as space,^26^ perceived relevance to readers^27^ and economics.^28^ The way in which various media select and package stories highlights certain aspects and shapes understandings around problem definition, moral judgements and potential solutions.^29 30^

Given this, we wished to identify how UK news media represented smoking and smoking bans in prisons over a period during which total smoking bans were introduced in some English and Welsh (E&W) prisons. We were particularly interested in news media available to prisoners, prison staff and policy-makers in Scotland, where smoke-free prisons were being discussed, but firm policy intentions were not established^i^. We therefore conducted a thematic analysis to investigate representations of smoking and smoking bans in prisons within UK/Scottish newspapers and broadcast media over a 17-month period during 2015–2016.

## Methods

### Media and search period selection

Our sample consisted of 12 national (UK/Scottish) daily and corresponding Sunday newspapers (total n=21). These represented all national print titles consistently available in Scotland throughout the search period.^31^ They also represented three genres/readerships: nine ‘popular’ tabloids (generally less serious, more sensationalist); four ‘middle-market’ tabloids and eight ‘serious’ (diverse politically, generally more socioeconomically advantaged readership) newspapers. In addition, since the UK press is politically polarised, they represented both right-leaning (particularly the Daily Mail/Sunday Mail and Daily Telegraph/Sunday Telegraph) and left-leaning (particularly the Daily Mirror/Sunday Mirror and Guardian/Observer) newspapers. This typology has been used in other analyses of UK print media discourses around tobacco, health-related and non-health-related issues to select a broad newspaper sample representing various readership profiles and political orientations.^25 32–38^ We used Scottish editions of UK national publications (almost identical to versions sold elsewhere in the UK, but with a Scottish slant) where archives of publications were available and also searched 43 Scottish local newspapers published within 100 miles of a Scottish prison. We also searched news programme transcripts of three television channels (BBCNews24, SKY News, BBC Scotland) and two speech radio stations (BBC Radio 4, BBC Radio 5 Live). While not exhaustive, we aimed to sample a diverse range of media to which prison staff, prisoners and policy-makers may have access. Online-only content was excluded since UK prisoners do not routinely have internet access.

The search period 1 January 2015 (note: UK date format throughout) to 1 June 2016 was selected to include a time when smoke-free prison policy was implemented in several countries, including all Welsh and some English prisons.

### Search strategy

We searched for potentially relevant news pieces using eight distinct searches (four search term combinations across two search fields) in the electronic database Nexis. Search terms related to smoking and/or e-cigarettes and/or secure institutions and/or/not smoking bans (online supplementary 1). These identified 3302 newspaper articles/broadcast transcripts (hereafter articles/broadcasts; 2256 national newspapers, 287 local Scottish publications, 759 broadcast). Articles/broadcasts were screened (by AR, with random sampling checks (n=50) by HS/KH) following the process shown in online supplementary 2, and inclusion/exclusion criteria detailed in box 1. Table 1 shows the number of eligible articles/broadcasts by media outlet/genre (90 articles; 16 broadcasts).

Box 1 **Inclusion and exclusion criteria**

**Inclusion criteria**

Contemporary presentations of:

- smoking in prisons
- smoke-free prisons or
- prisons’ exemption from smoke-free legislation.

**Exclusion criteria**

- Web-only content or
- Concerned with:
  - historical (>50 years) references to smoking in prison
  - smoking of cannabis or other illegal substances in prison
  - foreign cases where no UK comparisons were made or could be drawn or
  - minor mentions of cigarettes in wider contexts of illicit drug taking.

**Basis on which most articles/broadcasts excluded**

Focused around:

- cigarette packaging;
- use of e-cigarettes in hospital grounds;
- legislation banning smoking in cars with children;
- illicit drugs within and outside of prisons;
- smoking cessation, not specific to prison context;
- tobacco industry and regulation;
- criminal cases;
- unrelated articles picking up search language, for example, music review, sport reviews, food columns or international politics.

10.1136/tobaccocontrol-2017-053868.supp1 Supplementary data

10.1136/tobaccocontrol-2017-053868.supp2 Supplementary data

**Table 1:** Included newspaper articles and broadcasts and numbers with each broad topic/focus

News outlet and genre	Abbreviation used in text	Total articles	Constructs of prison/prisoners including tobacco-related culture	Legal cases (UK Black, Gage, Guild; French)	Anticipation/announcement of E&W smoke-free prisons	Ravenhall riot/Australian smoke-free prisons	General re tobacco/e- cigarette smoking (bans), mention of prisons	Prison secondhand smoke levels and risks	E-cigarettes in E&W prisons	E&W smoke-free prisons incident
Newspaper										
‘Popular tabloid’—av words 223		**32**	**14**	**10**	**2**	**1**	**0**	**2**	**2**	**1**
*The Sun*	Su	17	10	5	1	1				
*Daily Mirror*	DM	10	3	3	1			1	1	1
*Daily Star of Scotland*	DS	2	1	1						
*The People*	Pe	2						1	1	
*Daily Record* (Scottish national)	DR	1		1						
‘Middle-market’ tabloid—av words 233		**11**	**7**	**3**	**0**	**0**	**0**	**1**	**0**	**0**
*Scottish Daily Express*	Ex	7	5	2						
*Scottish Daily Mail*	DM	3	2	1				1		
‘Serious’ newspaper —av words 657		**42**	**8**	**12**	**5**	**9**	**5**	**2**	**0**	**1**
*Guardian*	Gu	16	3	2	2	7	2			
Times	Ti	10	1	5	1		1	1		1
*Daily Telegraph*	DT	5	1	2	1	1				
*Herald* (Scottish national)	He	4	1	2		1				
*Sunday Times*	ST	3	2				1			
*Observer* (Sunday paper)	Ob	2			1			1		
*Scotsman* (Scottish national)	Sc	2		1			1			
Scottish local— av words 342		**5**	**2**	**1**	**0**	**0**	**2**	**0**	**0**	**0**
*Evening Times* (Glasgow)	ET	2		1			1			
*Aberdeen Press & Journal*	AP	1	1							
*Carrick Gazette*	CG	1					1			
*Edinburgh Evening News*	Ee	1	1							
Broadcast										
TV—av words 365		**11**	**0**	**5**	**6**	**0**	**0**	**0**	**0**	**0**
BBC News 24	BBCNews24	8		5	3					
SKY News	SKYNews	2			2					
BBC 1 Scotland	BBCScot	1			1					
Radio—av words 846		**5**	**1**	**0**	**4**	**0**	**0**	**0**	**0**	**0**
BBC Radio 4	BBCRad4	4			4					
BBC Radio 5 Live	BBCRad5L	1	1							
Total		106	32	31	17	10	7	5	2	2

E&W, English and Welsh.

### Analysis

As a first level of description, we categorised each article/broadcast in terms of overall broad topic/focus as indicated via either its headline or a single reading. This was an inductive process, conducted completely separately by AR/HS with complete agreement once slight variations in one category (‘Constructs of prisons/prisoners including tobacco-related culture’, initially broken down into three subcategories by AR and two by HS) were resolved by collapsing into a single category. This exercise resulted in eight mutually exclusive categories, four covering the bulk (90/106) of the articles/broadcasts: *constructs of prisons/prisoners including tobacco-related culture*; *legal cases*; *anticipation/announcement of the move to smoke-free prisons in England and Wales;* and *Ravenhall riot/Australian smoke-free prisons* (online supplementaries 3 and 4 detail each article/broadcast and broad topic/focus category). These categories allowed us to summarise the coverage overall and by media outlet/genre.

Our main analytical method was inductive thematic analysis, a subjective process conducted to identify the latent (less directly observable) content.^37 39^ All authors independently developed a draft coding frame over a series of stages, with AR reading all and, between them, HS/KH almost all (95%) of articles. Iterative discussion and several stages of exploratory double/triple coding finalised the coding frame and identification of four broad thematic categories: smoking in prisons; smoking bans in prison; broader aspects and country of reference. (Online supplementary 5 shows themes and subthemes.) Further triple coding of 25 articles/broadcasts resulted in only minor disparities between coders. Subsequent small amendments and triple coding/discussion of five articles resulted in full agreement. Final coding was undertaken using NVivo V.11 software, and written summaries of thematic categories were produced (AR, HS). There was some overlap between our initial broad themes, and further analysis identified subthemes which we report on below: *constructs of prisoners and prison smoking cultures* (using material coded in subthemes under ‘smoking in prisons’ and ‘broader issues’); *smoke-free rights and the freedom to smoke* (subtheme under ‘smoking bans in prisons’); *SHS and health* (subtheme under ‘smoking in prisons’) and *smoke-free prisons: (anticipated) consequences and preparatory actions* (subthemes under ‘smoking bans in prisons’ and ‘broader issues’).

10.1136/tobaccocontrol-2017-053868.supp5 Supplementary data

## Results

### Events and coverage summary

**Figure 1:**
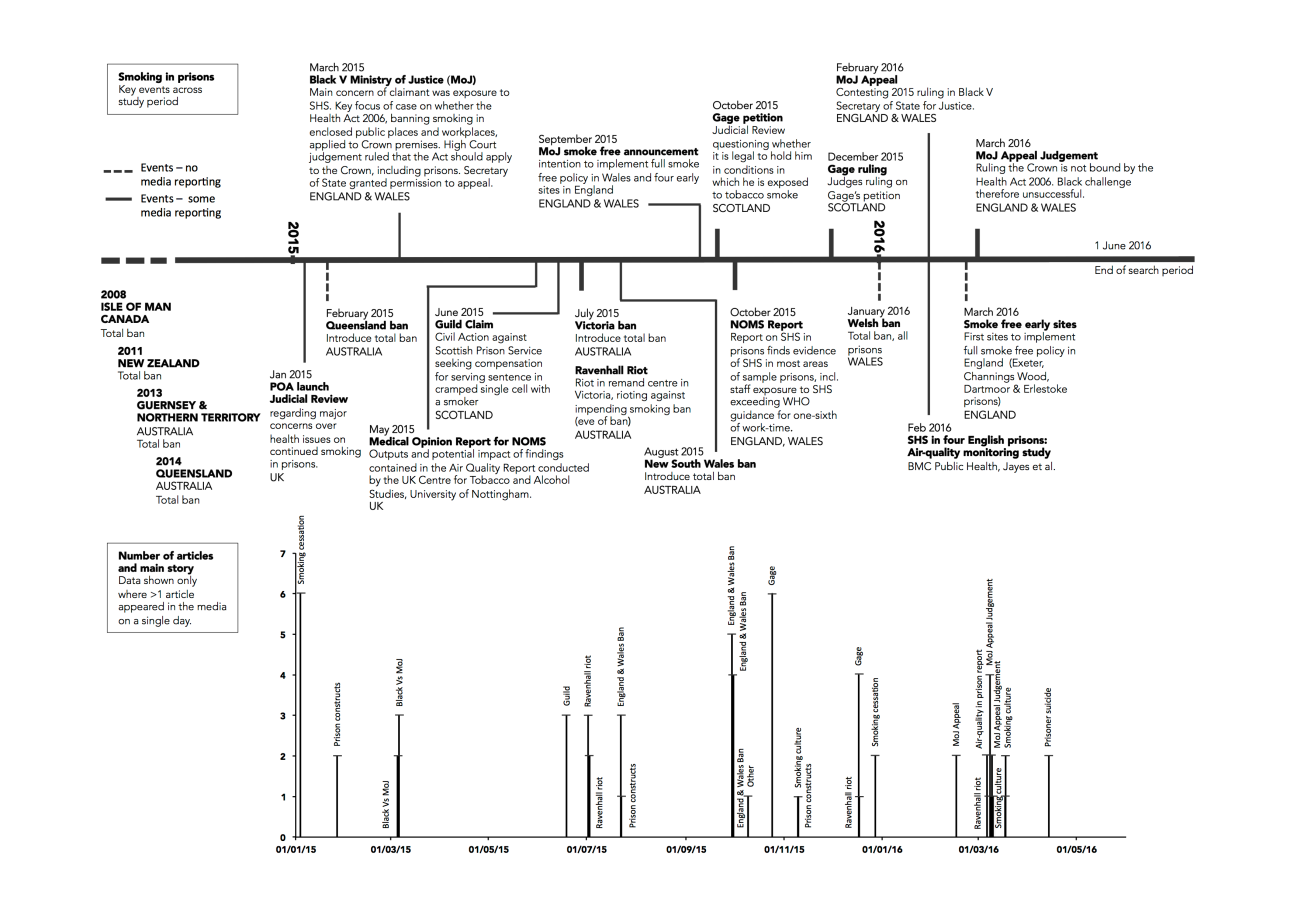
Timeline. MoJ, Ministry of Justice; SHS, secondhand smoke.

Several relevant events occurred during the search period (figure 1). Smoke-free prison policy was implemented in several Australian states,^40^ all Welsh prisons (January 2016) and four English early adoption sites (March 2016), following a UK Ministry of Justice (MoJ) announcement on 29 September 2015 (hereafter MoJ announcement).^41^ A small number of incidents were directly/indirectly linked to prison smoking, including: rioting at an Australian remand centre (Ravenhall riot) the day before implementation of a ban (June 2015) and a Welsh prisoner suicide (April 2016). Two studies reported SHS levels in E&W prisons,^11 12^ and there were three reported legal cases around the rights of non-smoking prisoners in England (Black^ii^) and Scotland (Guild^iii^, Gage^iv^). In March 2015, Black won a ruling that the 2006 E&W Act banning smoking in public places applied to state prisons; in March 2016, the MoJ won an appeal against this. The Guild claim (June 2015) involved a non-smoking ex-prisoner seeking compensation for sharing a cell with a smoker, and Gage (October 2015) took the Scottish Government to court for failing to protect him from SHS; in December 2015, it was announced he had lost his case. Figure 1 shows the number of articles/broadcasts, by date, highlighting clustering round events.

Online supplementary tables 3 and 4 show chronologically: newspaper, genre, word count, headline and broad topic/focus for each article; and channel/station, word count, description and broad topic/focus for each broadcast. Table 1 highlights that coverage was concentrated in just five outlets: two ‘popular’ tabloids (*Sun*, n=17 articles; *Daily Mirror*, n=10, two ‘serious’ newspapers (*Guardian*, n=16; *Times*, n=10) and BBCNews24 (n=8 broadcasts). Only 5 of 282 articles from Scottish local publications screened were selected for inclusion as containing any relevant content. Table 1 also shows over half (57/90) the articles overall, and three-quarters (34/43) in tabloids, focused on legal cases and various aspects of prisoners’ lives. Coverage of smoking bans in prisons was greater in the serious newspapers, due to more reporting extensively and repeatedly on the Ravenhall riot (largely in the *Guardian*) than on the MoJ announcement. In contrast, two-thirds (10/16) of the broadcast media focused on the MoJ announcement. Table 1 also highlights the relatively low word count of most coverage. Articles tended to be short; 14 were below 100 words and only 7 were over 1000. In the broadcast media, several news items were around 50 words (around 25 s, based on average reading speed; http://www.speechinminutes.com/), and only two longer discussions around the MoJ announcement were over 1000 words.

### Qualitative content analysis

#### Constructs of prisoners and prison smoking cultures

Table 2 shows representation of the themes within the articles/broadcasts. Over half (57/106) were coded as including material related to prisoners and prison smoking cultures which was more likely to feature in tabloids rather than ‘serious’ newspapers.

**Table 2:** Included newspaper articles and broadcasts and numbers mentioning each theme

News outlet and genre	Total articles*	Constructs of prisoners and prison smoking culture	Smoke-free ‘rights’ and freedom to smoke		SHS and health			Anticipated unrest and violence re smoke-free prisons
			Prison staff and a smoke-free workplace	Prisoners (usually associated with legal cases)	Prison staff health	Prisoner health	Health impacts of SHS	
Newspaper								
‘Popular tabloid’	**32**	**20**	**0**	**10**	**5**	**4**	**4**	**14**
*The Sun*	17	12	–	6	2	1	4	4
*Daily Mirror*	10	6	–	2	1	1	–	6
*Daily Star of Scotland*	2	2	–	1	1	1	–	2
*The People*	2	–	–	–	1	1	–	1
*Daily Record* (Scottish national)	1	–	–	1	–	–	–	1
‘Middle-market tabloid’	**11**	**11**	**0**	**4**	**4**	**1**	**1**	**6**
*Scottish Daily Express*	7	7	–	3	3	1	1	3
*Scottish Daily Mail*	4	4	–	1	1	–	–	3
‘Serious’ newspaper	**42**	**16**	**6**	**14**	**13**	**9**	**6**	**23**
*Guardian*	16	5	3	2	5	4	2	11
Times	10	3	–	5	3	2	4	3
*Daily Telegraph*	5	1	1	2	2	–	–	4
*Herald* (Scottish national)	4	2	–	2	1	1	–	3
*Sunday Times*	3	2	–	–	–	–	–	–
*Observer* (Sunday paper)	2	2	2	1	2	2	–	2
*Scotsman* (Scottish national)	2	1	–	2	–	–	–	–
Scottish local	**5**	**2**	**0**	**1**	**1**	**0**	**0**	**1**
*Evening Times* (Glasgow)	2	–		1	1	–	–	1
*Aberdeen Press & Journal*	1	1	–	–	–	–	–	–
*Carrick Gazette*	1	–	–	–	–	–	–	–
*Edinburgh Evening News*	1	1	–	–	–	–	–	–
Broadcast								
TV	**11**	**5**	**1**	**7**	**4**	**4**	**0**	**11**
BBC News 24	8	4	1	6	2	2	–	5
SKY News	2	1	–	–	1	1	–	2
BBC 1 Scotland	1	–	–	–	–	–	–	1
Radio	**5**	**3**	**0**	**1**	**1**	**1**	**0**	**3**
BBC Radio 4	4	2	–	1	1	1	–	3
BBC Radio 5 Live	1	1	–	–	–	–	–	–
Total*	106	57	7	36	27	18	11	55

*Total articles not equivalent to total ‘mentions’ since some covered more than one theme.

SHS, secondhand smoke.

Prisoners were frequently presented as unpredictable and disruptive. Headlines and leading sentences commonly constructed a negative view of prisoners, particularly in the tabloids and when reporting legal cases: ‘*caged killer*’ (Su/241015), ‘*moaning crook*’ (DS/180615). There were frequent references to taxpayers’ costs and/or prison as a *‘skoosh’* ^v^ (Ex/260115); and of prisoners as an undeserving population, highlighted particularly in several articles around mindfulness courses for prisoner smoking cessation.

A distinct prison smoking culture was portrayed. Articles/broadcasts reported high prisoner smoking rates and presented cigarettes as essential everyday items, an ‘*emotional prop*’ (BBCRad4/290915), the only ‘*legal stimulant*’ (Gu/050315) and currency for anything from chocolate to sex. A few articles attempted to explain high smoking rates, linking smoking with poor mental health and noting long waiting lists for smoking cessation programmes.

#### Smoke-free rights and the freedom to smoke

As table 2 shows, there was far more coverage of prisoners’ smoke-free rights (usually associated with legal cases) than staff rights to a smoke-free workplace. Indeed, staff rights were only raised in ‘serious’ newspapers and one TV news broadcast.

Reporting of the Black versus MoJ case focused primarily on poorly enforced prison smoking restrictions and SHS. Three articles included comments from Black’s counsel describing it as ‘*disappointing*’ that ‘*non-smoking prisoners and prison staff are [denied] the same legal protection from the dangers posed by SHS as the rest of us*’ (Ob/180715; Gu/080316; DT/090316). Most articles covering the Guild case included his legal statement that sharing a single-person cell ‘*breaches human right*s’ (Sc/170615; Su/180615; DS/180615), rather than focusing on smoke-free-related rights. Coverage of Gage’s case described his wishes to be moved to a smoke-free prison section due to his fear of lung cancer. One article noted the case was lost because his current circumstances, ‘*a relatively modern prison with ventilation systems*’ (Ti/171215), were deemed not unreasonable.

Freedom for prisoners to smoke was referred to in a small number of *Guardian* articles on the Ravenhall riot. These noted the balance between perceived psychological benefits of smoking to the smoker versus minimal health risks to others if smoking occurred outdoors, and quoted various Australian academics, members of parliament and activists in respect of the cruelty of removing the legally protected ability to smoke from a group with few entitlements (Gu/010715; Gu/060715; Gu/150715). Unlike the right to smoke-free environments, smoking is not a human right, but is a legally protected ability in certain locations. However, two articles covering a Welsh prisoner suicide included quotes from another prisoner’s partner specifically referring to prisoner-smoker ‘rights’ ‘*He told staff he was going to do it. It’s human rights they should be allowed to smoke*’ (Mi/130416; Ti/130416).

Articles covering the MoJ announcement made little mention of freedom to smoke or rights to a smoke-free environment. This contrasted with several broadcasts which discussed staff rights to a smoke-free workplace versus prisoner-smoker freedoms. These included a prison officer describing it as ‘*disgraceful*’ that they were *‘the only workers in Great Britain not protected by (smoke-free) legislation*’ (BBCNews24/180115) and a former prisoner calling smoking ‘*the last thing that prisoners have got that is legal*’ (SKYNews/301015).

#### SHS and health

Table 2 shows that there was little specific discussion of the health impacts of SHS within the coverage, and a greater focus on staff than prisoner health. Apart from the complete lack of specific discussion of the health impacts of SHS in the broadcast media, there was no clear patterning in health theme codings by genre.

Most references to health appeared in relation to exposure of prison staff and/or prisoners to SHS when reporting legal cases. References early in the search period were frequently aligned with MoJ/Prison Service/court comments rejecting the severity of claimed SHS impacts, and a few articles describe MoJ ‘*hindering*’ publication of study reports. Later, particularly after the MoJ announcement, references suggested Prison Service recognition of SHS risks to prisoners and staff.

Although a quarter (n=27) of articles/broadcasts alluded to concern for prison staff health, references were often vague. Most (apart from two reporting on miscarriage/stillbirth as a specific risk) simply referred to SHS exposure ‘*exceeding*’ WHO limits or being ‘*off the radar*’ (BBCNews24/180115). However, prison staff safety was frequently mentioned, usually explicitly associated with concern over prisoners’ (violent) reactions to introducing smoke-free policy.

Reference to prisoner health occurred primarily in the context of the legal cases, noting ‘*claims*’ SHS might exacerbate existing health problems (Black) or increase cancer risk (Gage). Only one article (Mi/060316) specifically focused on prisoner health. However, prisoner health also received some mentions alongside that of prison staff in reporting of SHS studies. A few *Guardian* articles mentioned ‘*duty of care to help smokers, no matter who they are or where they are, to quit*’ (Gu/060715; Gu/150715; Gu/081015).

#### Smoke-free prisons: (anticipated) consequences and preparatory actions

Reporting of the legal cases, Ravenhall riot and MoJ announcement almost universally included negative (anticipated) consequences of smoke-free prison policies, particularly warnings of unrest, violence or instability. As table 2 shows, half (n=55) the articles/broadcasts were coded as including these concerns, which were evident regardless of media outlet/genre. Most made no reference to why or how any such fears could be addressed.

As an example among the legal coverage, the Black case, which began early in the search period, routinely incorporated comments from authority figures around prisoners’ presumed reactions to smoke-free policies. This included the Judge who suggested ‘*prisoners who feel the need to smoke may be resistant to the criminalising of that conduct*’ (Gu/050315, DT/060315, Su/060315, Ob/180715).

Several *Guardian* articles included detailed description of the Ravenhall riot: 300 ‘*prisoners with faces covered and carrying weapons*’ (Gu/300615), in a disturbance which took 15 hours to contain, causing ‘*$25m*’ damage (Gu/171215) and minor injuries to staff and prisoners (Gu/010715). All coverage attributed it to the imminent ban: ‘*Inmates stage riot after new cigs ban*’ (Su/020715). Two *Guardian* articles included comments from Australian researchers linking ‘*removal of a coping mechanism*’ with ‘*increased levels of tension and risk of aggression and violent responses*’ (Gu/060715, Gu/150715).

Reporting on the MoJ announcement, which occurred 3 months after Ravenhall, also included warnings: ‘*Jail unrest feared over smoking ban plans*’ (Ob/180715); ‘*risk of flare ups as lags* ^vi^ *battle withdrawal*’ (Su/300915). Fear of riots and instability were presented as reasons for delaying decisions around implementation of smoke-free prisons (Ob/180715; Ob/260715) and the rationale for a phased approach to implementation. A *Sun* article covering the MoJ announcement referred back to Ravenhall: ‘*In July hundreds of inmates rioted for 15 hours at a prison in Melbourne, Australia, after a smoking ban was brought in*’ (Su/300915). Widely quoted sources emphasised priorities and concerns, from a Government statement around ‘*operational safety and security of prisons*’ (Ti/300915) to an ex-prisoner suggesting ‘*if this ban comes in, there will be violence. Trust me*’ (SKY News/301015).

In addition to unrest, some reporting in anticipation of/around the MoJ announcement described other negative potential outcomes of smoke-free prisons, including concerns tobacco would be substituted in various ways, smuggled or traded ‘*like other banned substances*’ (BBCRad4/220715, Ti/300915) with ‘*inmates using tobacco as money*’ (BBCScot/220715). A few articles, including one in the *Sun* headlined ‘*£11 million patches and E-cigs for Lags*’ (Su/300915), mentioned costs to the public purse. However, none mentioned potential savings resulting from reduced smoking-related illness.

Despite such alarmist representations, direct criticisms of smoke-free prison policy were less apparent. However, only the *Guardian* and *Observer* presented broader views on the implications of a ban, describing ‘*mixed succes*s’ (Gu/300615; Gu/010715) or noting ‘*Prisons all around the world have gone smoke-free with few problems*’ (Ob/260715). A detailed *Guardian* article unusually covered the successful smoking ban in Guernsey prison (Gu/081015), quoting both the Prison Governor describing the E&W ban as ‘*well overdue*’, dismissing ideas that trouble could follow, and a prisoner describing the health and financial benefits of being smoke-free. Notably, few other articles/broadcasts commented on the health benefits of smoke-free prisons.

Concerns around negative consequences were associated, in some coverage, with descriptions of specific preparatory actions for smoke-free prisons. These included the provision of stop-smoking information, counselling, nicotine alternatives and additional recreational activities as preparation for implementation of smoke-free prisons in Australia (Gu/010715; Gu/020715; Gu/060715; Gu/150715). Coverage of the MoJ announcement included the *Sun* noting provision of ‘*security-vetted e-cigarettes which convicts can buy with their own money instead of fags* ^vii^ *and tobacco*’ (Su/300915) while the *Guardian* described staff and prisoner engagement, opportunities to work, increased activities and the use of prison-tailored e-cigarettes in Guernsey’s smoke-free prison (Gu/081015). Overall, however, there was limited coverage around the need for appropriate cessation support (BBCNews24/180116; BBCRad4/290915; Gu/081015).

The only reports (two articles) of any incident apparently resulting from the E&W ban appeared in April 2016, following a prisoner suicide in Wales. Both mentioned other prisoners attributing this to insufficient NRT following smoke-free implementation, for example, ‘*Prisoner kills himself over jail ban on cigarettes*’ (Mi/130416). This coverage also included suggestions that ‘*tensions were running high over the ban*’, alongside prison officer denials of ‘*widespread unrest*’ (Ti/130416) and accounts of normal operations.

## Discussion

We analysed UK/Scottish media coverage of smoking in prisons and smoke-free prisons over 17 months during 2015–2016, when: smoke-free prison policies began to be introduced in E&W and discussed in Scotland; reports on SHS levels in E&W prisons were released; and there were three reported UK legal cases around prisoners’ smoke-free rights. Coverage was both relatively infrequent, comprising only 106 articles/broadcasts over the search period, and, generally, brief. As such it is unlikely to have significantly highlighted the topic for people unaware of the issues. Reporting in the left-leaning ‘serious’ *Guardian*/*Observer* (sister papers) was unusual. It included more in-depth coverage of the issues and themes, and more positive and detailed representations of smoke-free prisons.

Reporting largely reinforced a negative view of prisoners in discussions of rights to a smoke-free environment, prison smoking cultures and SHS. The general lack of explicit discussion of health impacts, particularly for prisoners, was notable. Reporting in anticipation of smoke-free prisons emphasised problems, despite lack of evidence of disorder resulting from smoking bans^2 19 20^; the only actual incidents covered were an Australian riot (repeatedly covered) and a suicide in Wales, attributed to lack of cessation support in a smoke-free prison. Anticipated negative outcomes, particularly violence and unrest, were consistently presented alongside discussion of future smoke-free policies, and when these were countered with more positive statements, references were vague and much shorter. The only representation of a largely trouble-free implementation of smoke-free prison policy was presented as unusual.

As in previous media analyses, stories were episodic, focused mainly on individuals or events, rather than broader public health or societal issues.^29 42^ Opportunities for health gains, and tackling smoking in one of the most disadvantaged populations in whom smoking remains high, were rarely presented, nor was health protection highlighted as a rationale for smoke-free prisons. Discussion of health inequalities in relation to smoking and the challenges of supporting smoking cessation in this population group were also missing.

This is the first study to explore representations of smoking in prisons and smoke-free prisons in news media and, as with similar analyses, ours is limited to selected news media. Unlike most similar studies,^25 26 43^ we complemented a wide national/metropolitan newspaper sample with a search of local (Scottish) publications. However, it is possible that had we included publications local to the few E&W prisons that implemented smoke-free prison policies in 2016, we might have identified more material. Our decision to exclude online-only news media because it was unavailable to prisoners means we omitted otherwise widely accessible coverage, although some might have seen such coverage before prison entry or via contact with people ‘outside’ or prison staff. It is likely that prisoners, prison staff and policy-makers engage with different media outlets/genres; for example, policy-makers may have seen more ‘serious’ newspaper coverage. However, this was not examined in our study. Although our analysis attempted to capture the complex and sometimes mixed messages in the material, another limitation is that our study, like others,^25^ was not designed to examine audience reception.

We suggest that while infrequent, any news media representations of prisoners (in negative terms), smoking in prisons (as ‘normal’, embedded, everyday) and the introduction of smoke-free prisons (as associated with unrest and difficulties) will tend to impact negatively on the views of stakeholders about smoke-free prison policies. We recommend that when engaging with the media on this issue, policy-makers, prison service managers and tobacco control advocates aim to counter this via provision of accurate facts around the generally smooth transitions around the world to date. We also suggest that they emphasise opportunities to impact on the health of both one of the few employee groups to be exposed to SHS in many countries, and one of the most disadvantaged societal groups with very high smoking rates.

**What this paper adds**

- This is the first study internationally to explore the representation of smoking in prisons and smoke-free prisons in national and local news media.
- It identified infrequent reporting relating to smoking in prisons or smoke-free prison policies and little in-depth engagement with the issues.
- Coverage tended to reinforce a negative view of prisoners, avoid explicit concern for prisoner or prison staff health and largely ignored the health gains of smoke-free policies.
- Presumed unrest and instability were frequently linked to the introduction of smoke-free prisons, despite general lack of evidence.

10.1136/tobaccocontrol-2017-053868.supp3 Supplementary data

10.1136/tobaccocontrol-2017-053868.supp4 Supplementary data

## Footnotes

i Note that the Scottish Prison Service announced on 17 July 2017 that its intention was for all prisons in Scotland to be smoke free by November 2018.

ii Black v Secretary of State for Justice (2015) C0/1258/2014, 5 March https://www.judiciary.gov.uk/wp-content/uploads/2015/03/black-v-justicefinal.pdfSecretary of State for Justice v Paul Black (2016) C1/2015/1018, 8 March https://www.judiciary.gov.uk/wp-content/uploads/2016/03/secretary-of-state-for-justice-v-paul-black.pdf

iii Legal/court records unavailable via web search. http://ash.org.uk/media-and-news/ash-daily-news/ash-daily-news-for-18-june-2015/

iv Opinion of Lord Armstrong in the petition of William Lewis Gage for Judicial Review of his detention by the Scottish Ministers in conditions in which he is exposed indoors to environmental tobacco smoke. (2015) CSOH 174, 16 December. https://www.scotcourts.gov.uk/search-judgments/judgment?id=b08dfca6-8980-69d2-b500-ff0000d74aa7

v Skoosh=Scottish word meaning an easy task.

vi Lags=UK informal term for prisoners.

vii Fags=UK informal term for cigarettes.
