# Supplementary material for: UK news media representations of smoking, smoking policies and tobacco bans in prisons
Source: Tob Control. 2018 Feb 19;27(6):622–30. doi: 10.1136/tobaccocontrol-2017-053868 (PMC6252368; doi:10.1136/tobaccocontrol-2017-053868)
Supplement: Supplementary data [file tobaccocontrol-2017-053868supp001.pdf]

## Supplementary 1 – Media searched and search terms used

| Media searched using Nexis | Search field | Search terms[1] |
|---|---|---|
| **Newspapers**<br><br>Searches 1,2, 3, 4 - a & b<br><br>National UK/Scottish daily and corresponding Sunday publications (where published and available though archive database): n=21<br><br>The Sun (English with occasional Scottish edition), The Sun on Sunday, The Mirror, The Sunday Mirror, Scottish Star, Scottish Sunday Star, The People, Daily Record, Scottish Express, Scottish Sunday Express, Scottish Daily Mail, Scottish Mail on Sunday, The Guardian, The Observer, The Times, The Sunday Times, The Daily Telegraph, Sunday Telegraph, Herald, The Sunday Herald, The Scotsman, Scotland on Sunday.<br><br>Local Scottish: all local publications held by LexisNexis with a publication office within 100 miles of a Scottish prison: n=43<br><br>**Broadcast:**<br><br>Searches 1,2, 3, 4 – b[2]<br><br>BBC News 24,<br>BBC Radio 4,<br>BBC Radio 5 Live,<br>BBC1 Scotland,<br>SKY News | a. At the start (headline and leading paragraph)<br>b. Three or mentions | (1)<br>smok! OR tobacco OR cig! OR ecig! OR nicotine OR e-cig! OR electronic cig! OR vape! OR vapo!<br>AND<br>Ban! OR legislation OR regulation! OR Bill<br>AND<br>prison! OR jail! OR incarcerate! OR inmate! OR criminal! OR offender! OR justice!<br><br>(2)<br>smok! OR tobacco OR cig! OR ecig! OR nicotine OR e-cig! OR electronic cig! OR vape! OR vapo!<br>AND<br>Ban! OR legislation OR regulation! OR Bill<br>AND<br>bully! OR contraband OR black-market! OR black market! OR quit! OR cessation OR patch! OR human right!<br><br>(3)<br>smok! OR tobacco OR cig! OR ecig! OR nicotine OR e-cig! OR electronic cig! OR vape! OR vapo!<br>AND<br>prison! OR jail! OR incarcerate! OR inmate! OR criminal! OR offender! OR justice!<br>NOT<br>Ban! OR legislation OR regulation! OR Bill<br><br>(4)<br>smok! OR tobacco OR cig! OR ecig! OR nicotine OR e-cig! OR electronic cig! OR vape! OR vapo!<br>AND<br>bully! OR contraband OR black-market! OR black market! OR quit! OR cessation OR patch! OR human right!<br>NOT<br>Ban! OR legislation OR regulation! OR Bill |
| **Additional website search:**<br>SKY News[3] | Single word search using search function on website | Prison, smoke, cigarette, smoking |

[1] A further search including terms "detention cent!", "secure hospital!", and "secure psychiatric hospital!" was dropped after exploratory searches returned <10 articles all with no relevance to smoking in prisons or smoke-free prisons policies.

[2] Due to the way in which radio and television programmes are structured (usually with no story headline), broadcast transcripts were searched solely using search field b (three or more mentions). A number of test searches were carried out to ensure this would not limit findings.

[3] Additional searches carried out using SKY news website as database 'check' due to very low number of identified mentions. One duplicate and no additional mentions were found.
