# Supplementary material for: UK news media representations of smoking, smoking policies and tobacco bans in prisons
Source: Tob Control. 2018 Feb 19;27(6):622–30. doi: 10.1136/tobaccocontrol-2017-053868 (PMC6252368; doi:10.1136/tobaccocontrol-2017-053868)
Supplement: Supplementary data [file tobaccocontrol-2017-053868supp002.pdf]

Supplementary 2 – Search & screening chart

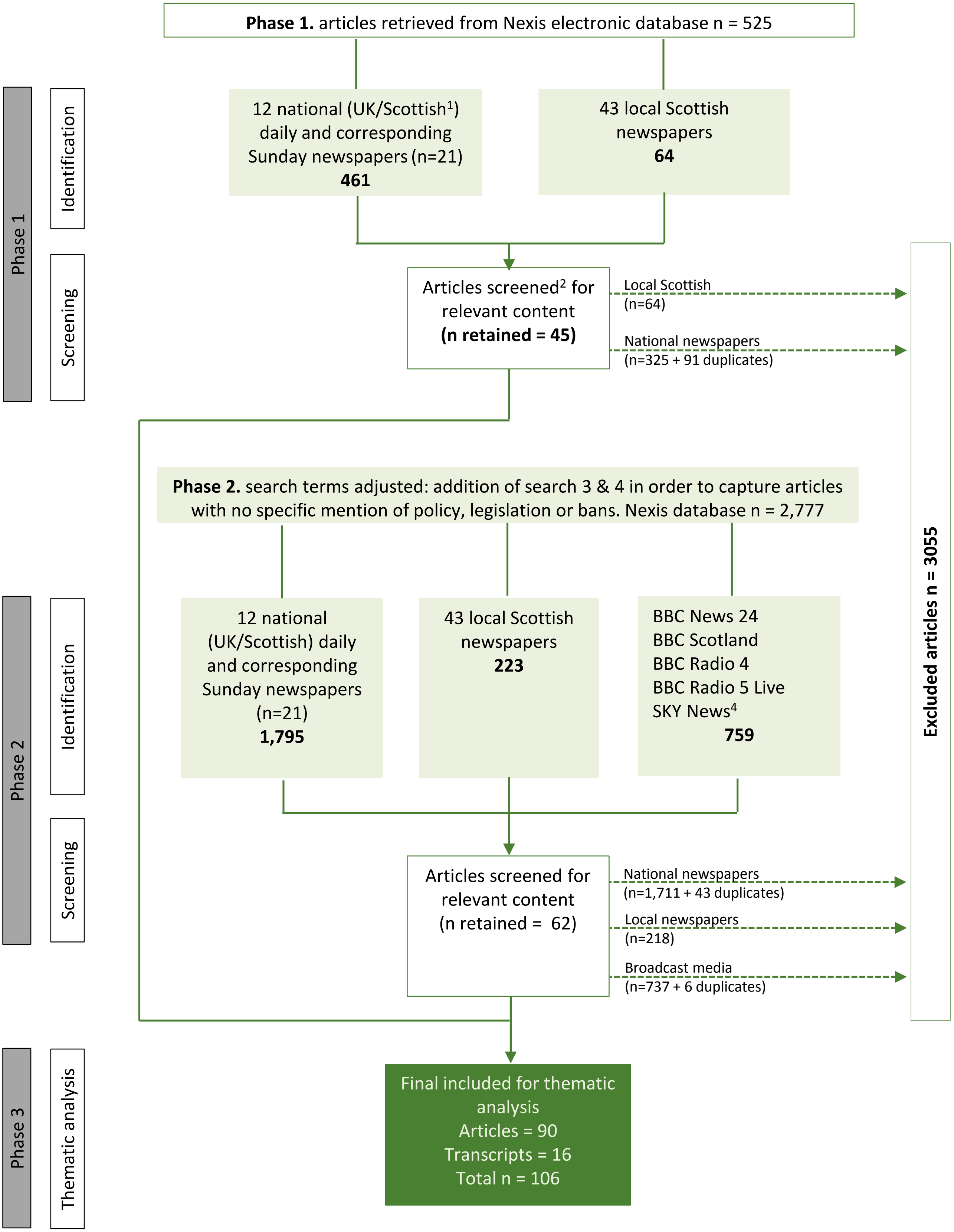

[1] Scottish editions of UK national publications were used where produced and available through archive database, see Supplementary 1
[2] See Table 1 inclusion criteria
[3] See Table 1 exclusion criteria
[4] Additional searches carried out using SKY news website as database 'check' due to very low number of identified mentions. One duplicate and no additional mentions were found.
