# Supplementary material for: UK news media representations of smoking, smoking policies and tobacco bans in prisons
Source: Tob Control. 2018 Feb 19;27(6):622–30. doi: 10.1136/tobaccocontrol-2017-053868 (PMC6252368; doi:10.1136/tobaccocontrol-2017-053868)
Supplement: Supplementary data [file tobaccocontrol-2017-053868supp005.pdf]

Supplementary 5 - Smoking and smoke-free prisons coding frame: themes and sub-themes

Smoking in prisons

- Health (e.g. prison staff; prisoners; second-hand smoke and air quality monitoring; compensation claims)
- Smoking culture in prison

Smoking bans in prisons

- Anticipated (e.g. violence; tobacco control/contraband issues; negativity towards bans; financial costs/savings; success)
- Implementation (e.g. preparation; timing; experiences; financial costs)
- Smoke-free rights and legal rulings around freedom to smoke
- Standpoint / frame of article or sources

Broader issues

- Constructs of prisoners / prison life
- 'Facts' (e.g. smoking rates; smoking association with mental health)
- Alternatives (e.g. nicotine replacement; e-cigarettes; illicit alternatives; cessation support)
- Prison smoking within broader tobacco control context
- Other

Country of reference

- England and Wales
- Scotland
- UK specified or not
- Australia
- Canada
- Guernsey
- Isle of Man
- New Zealand
- United States
- Other incl. Hong Kong, Romania, France
