# Supplementary material for: UK news media representations of smoking, smoking policies and tobacco bans in prisons
Source: Tob Control. 2018 Feb 19;27(6):622–30. doi: 10.1136/tobaccocontrol-2017-053868 (PMC6252368; doi:10.1136/tobaccocontrol-2017-053868)
Supplement: Supplementary data [file tobaccocontrol-2017-053868supp003.pdf]

Supplementary 3: Table showing articles – ordered chronologically throughout search period with newspaper, genre, ID, date, word count, headline and broad topic

Paper	Genre	ID	Date	N words	Article headline	Broad topic
Scottish Daily Mail	Middle market tabloid	DM123	03/01/2015	341	Buddhism lessons to help prisoners give up smoking	Constructs of prison / prisoners incl tobacco-related culture
Herald (Scottish national))	Serious newspaper	HE136	03/01/2015	147	Meditation for inmates who smoke	Constructs of prison / prisoners incl tobacco-related culture
Scottish Daily Express	Middle market tabloid	Ex142	03/01/2015	192	What bright spark came up with this prison plan?	Constructs of prison / prisoners incl tobacco-related culture
Scottish Daily Express	Middle market tabloid	Ex143	03/01/2015	335	Prisoners taught meditation to help them stop smoking	Constructs of prison / prisoners incl tobacco-related culture
Sun	Popular tabloid	Su154	03/01/2015	70	Lags free the mind	Constructs of prison / prisoners incl tobacco-related culture
Daily Star of Scotland	Popular tabloid	DS162	03/01/2015	273	Lags 'meditate' to get off cigs; Buddhist course for smokers in jail	Constructs of prison / prisoners incl tobacco-related culture
Daily Telegraph	Serious newspaper	DT158	10/01/2015	229	Carer 'killed so prisoner could keep smoking'	Constructs of prison / prisoners incl tobacco-related culture
Evening Times (Glasgow, Scotland)	Scottish local	ET166	19/01/2015	380	Council launches two-year plan to stub out smoking	General re tobacco / e-cigarette smoking (bans), mention of prison(ers)
Sun	Popular tabloid	Su021	25/01/2015	237	£20M bill for cons' cheap cigs & sweets: Prison tuck shops outrage	Constructs of prison / prisoners incl tobacco-related culture
Scottish Daily Express	Middle market tabloid	Ex141	26/01/2015	345	Prison tuck shops a good deal for jailbirds	Constructs of prison / prisoners incl tobacco-related culture
Scottish Daily Express	Middle market tabloid	Ex045	26/01/2015	115	Jail subsidy is all wrong;	Constructs of prison / prisoners incl tobacco-related culture
Guardian	Serious newspaper	Gu016	07/02/2015	2,147	Smoke and mirrors: the stealth approach that aims to put cigarettes out for good; Smoking's never been banned, and probably it never will be - but Bristol's move towards 'official tutting' is meant to achieve the same goal. So will it work?	General re tobacco / e-cigarette smoking (bans), mention of prison(ers)
Sun	Popular tabloid	Su024	02/03/2015	837	UK's inmates smoke and have TV...I make mine sleep in tents and bury the dead	Constructs of prison / prisoners incl tobacco-related culture

Paper	Genre	ID	Date	N words	Article headline	Broad topic
Guardian	Serious newspaper	Gu008	05/03/2015	580	Smoking ban must be enforced in prisons, judge rules; Justice secretary wrongly understood jails to be exempt, but is given time to appeal against the ruling and its implementation is postponed	Legal cases (Black - ruling that E&W ban on smoking in public places applies in prisons)
Sun	Popular tabloid	Su020	06/03/2015	94	Jail smoking ban	Legal cases (Black - ruling that E&W ban on smoking in public places applies in prisons)
Daily Mirror	Popular tabloid	Mi035	06/03/2015	203	Snout is out; Cigs ban in jail communal areas	Legal cases (Black - ruling that E&W ban on smoking in public places applies in prisons)
Daily Telegraph	Serious newspaper	DT040	06/03/2015	319	Smoking banned in jail, judge rules	Legal cases (Black - ruling that E&W ban on smoking in public places applies in prisons)
Sun	Popular tabloid	Su150	30/03/2015	130	Kingpin' lives it up in prison cell-files	Constructs of prison / prisoners incl tobacco-related culture
Sun	Popular tabloid	Su149	26/04/2015	235	Glitter's 3 prison minders; Perv's cushy life; Paedo hires lags as heavies	Constructs of prison / prisoners incl tobacco-related culture
Times	Serious newspaper	Ti031	04/05/2015	493	Pregnant prison officers in passive smoking alert	Prison second-hand smoke levels and risks
Scottish Daily Mail	Middle market tabloid	DM033	08/05/2015	41	From The Times this week: [...]	Prison second-hand smoke levels and risks
Sun	Popular tabloid	Su148	17/05/2015	218	Whacky Baccy; Tobacco bounty on head of rapist after rammy	Constructs of prison / prisoners incl tobacco-related culture
Evening News (Edinburgh, Scotland)	Scottish local	EN167	26/05/2015	408	Saughton siege prisoners took hostage to demand KFC	Constructs of prison / prisoners incl tobacco-related culture
Sun	Popular tabloid	Su147	31/05/2015	291	Transsexual killer 'paying cons for sex'; Jail Chiefs Probe Cell Claims	Constructs of prison / prisoners incl tobacco-related culture
Scotsman (Scottish national)	Serious newspaper	Sc159	17/06/2015	436	Ex-prisoner seeks damages for sharing cell with smoker	Legal cases (Guild case launched – compensation for sharing cell with smoke)

Paper	Genre	ID	Date	N words	Article headline	Broad topic
Scottish Daily Express	Middle market tabloid	Ex140	18/06/2015	182	Ex- convict sues over sharing his cell with smoker	Legal cases (Guild case launched – compensation for sharing cell with smoke)
Sun	Popular tabloid	Su146	18/06/2015	172	Pokey's far too smokey; Cell cigs compo claim	Legal cases (Guild case launched – compensation for sharing cell with smoke)
Daily Star of Scotland	Popular tabloid	DS163	18/06/2015	315	Lag's £2.5K claim over smokey cell; Ex-inmate wants a pay-off	Legal cases (Guild case launched – compensation for sharing cell with smoke)
Scottish Daily Express	Middle market tabloid	EX044	19/06/2015	113	Smoking ban should include prison cells; Letters	Constructs of prison / prisoners incl tobacco-related culture
Guardian	Serious newspaper	Gu007	30/06/2015	523	Prisoners riot at Melbourne's Ravenhall remand centre over smoking ban; Seven News helicopter films prisoners with faces covered and carrying weapons, in unrest apparently connected to prison smoking ban	Ravenhall riot / Australian smoke-free prisons
Guardian	Serious newspaper	Gu006	01/07/2015	996	Fire breaks out at Melbourne prison a day after riots over smoking ban; 'There is no risk to staff or prisoner safety,' Corrections Victoria spokeswoman says as firetrucks attempt to contain blaze	Ravenhall riot / Australian smoke-free prisons
Daily Telegraph	Serious newspaper	DT041	01/07/2015	66	Australian prisoners riot over smoking ban	Ravenhall riot / Australian smoke-free prisons
Herald (Scottish national)	Serious newspaper	He048	01/07/2015	59	Smoking ban sets off prison rioting	Ravenhall riot / Australian smoke-free prisons
Guardian	Serious newspaper	Gu013	02/07/2015	601	Former deputy police commissioner to lead inquiry into Melbourne prison riot; Kieran Walshe, widely recognised for his work responding to the Black Saturday bushfires, will lead an independent investigation	Ravenhall riot / Australian smoke-free prisons
Sun	Popular tabloid	Su019	02/07/2015	123	Inmates stage riot after new cigs ban	Ravenhall riot / Australian smoke-free prisons

Paper	Genre	ID	Date	N words	Article headline	Broad topic
Guardian	Serious newspaper	Gu005	06/07/2015	558	Victoria urged to rethink smoking ban in prisons after violent riot; Premier Daniel Andrews rules out removing the ban, saying 'you don't reward that sort of appalling behaviour'	Ravenhall riot / Australian smoke-free prisons
Guardian	Serious newspaper	Gu004	15/07/2015	724	NSW prisons prepare for smoking ban with Victorian riot fresh in the memory; NSW Justice Health says it expects a 'smooth transition' to non-smoking jails after preparing for the move for the past two years	Ravenhall riot / Australian smoke-free prisons
Observer	Serious newspaper	Ob047	18/07/2015	541	Jail unrest feared over smoking ban plans; Court ruling that Parc prison must go smoke-free triggers concern about reaction from inmates, 80% of whom smoke	Anticipation / announcement of E&W smoke-free prisons
Guardian	Serious newspaper	Gu110	22/07/2015	345	Prisoner freed from Wandsworth by mistake posts images of himself online; Ryan Byrne, convicted of armed robbery, has published pictures of himself next to police vans	Constructs of prison / prisoners incl tobacco-related culture
Observer	Serious newspaper	Ob046	26/07/2015	511	Whitehall 'knew about health risks' to prison warders as anti-smokers push for total ban; Fears of mass riots in jails if authorities tried to stub out smoking are misplaced, says anti-tobacco charity	Prison second-hand smoke levels and risks
Guardian	Serious newspaper	Gu014	15/08/2015	2,159	The criminal's alphabet; Can't tell your turtles from your hotplate hamsters? Wise up with this indispensable guide to prison slang	Constructs of prison / prisoners incl tobacco-related culture
Sunday Times	Serious newspaper	StI168	23/08/2015	484	E-cigarettes not anti-smoking aids, says HSE	General re tobacco / e-cigarette smoking (bans), mention of prison(ers)
Guardian	Serious newspaper	Gu015	02/09/2015	619	Council staff face work-hours smoking ban; Nottinghamshire county council says move would improve workers' health, slash sick leave and increase time spent working	General re tobacco / e-cigarette smoking (bans), mention of prison(ers)
Guardian	Serious newspaper	Gu012	30/09/2015	622	Prison smoking ban begins in 2016 despite fears of unrest; Smoking ban will be phased in across England and Wales from next year, with complete ban in Welsh prisons taking effect in January	Anticipation / announcement of E&W smoke-free prisons
Sun	Popular tabloid	Su017	30/09/2015	373	£11M Patches & E-Cigs for lags: Smoking ban in prisons	Anticipation / announcement of E&W smoke-free prisons
Times	Serious newspaper	Ti030	30/09/2015	396	Prison chiefs fear unrest over introduction of smoking ban	Anticipation / announcement of E&W smoke-free prisons
Daily Telegraph	Serious newspaper	DT039	30/09/2015	252	Prisoners face smoking ban at eight jails	Anticipation / announcement of E&W smoke-free prisons
Guardian	Serious newspaper	Gu011	08/10/2015	1,087	How did a jail in Guernsey ban smoking with no unrest? Ahead of the prison smoking ban in England and Wales, Guernsey's Les	Anticipation / announcement of E&W smoke-free prisons

Paper	Genre	ID	Date	N words	Article headline	Broad topic
					Nicolles has been helping its prisoners to live without tobacco for two years	
Daily Mirror	Popular tabloid	Mi132	09/10/2015	48	Lag's chokey 'too smokey'	Legal cases (French prisoner)
Daily Mirror	Popular tabloid	Mi037	09/10/2015	235	Inspiring Aldi Angel	Anticipation / announcement of E&W smoke-free prisons
Sunday Times	Serious newspaper	STi156	18/10/2015	1,245	Mr Cameron, there ought to be more lags in Whitehall;	Constructs of prison / prisoners incl tobacco-related culture
Sun	Popular tabloid	Su151	19/10/2015	272	HMP Fight Club; Lawless prisons exposed; Cocky lags film punch-ups in cell	Constructs of prison / prisoners incl tobacco-related culture
Times	Serious newspaper	Ti119	24/10/2015	221	Hitman goes to court over smoky jail	Legal cases (Gage case launched – for protection from drifting second-hand smoke)
Scottish Daily Mail	Middle market tabloid	DM122	24/10/2015	602	Murderer's legal fight over passive smoking in prison cell	Legal cases (Gage case launched – for protection from drifting second-hand smoke)
Herald (Scottish national)	Serious newspaper	HE137	24/10/2015	493	Hitman lifer wants judicial review on passive smoking	Legal cases (Gage case launched – for protection from drifting second-hand smoke)
Daily Record (Scottish national)	Popular tabloid	DR	24/10/2015	258	Assassin sues over jail smoke	Legal cases (Gage case launched – for protection from drifting second-hand smoke)
Scottish Daily Express	Middle market tabloid	Ex139	24/10/2015	128	Killer in passive smoking court plea	Legal cases (Gage case launched – for protection from drifting second-hand smoke)
Sun	Popular tabloid	Su145	24/10/2015	163	Killer in jail cig bid; passive smoking	Legal cases (Gage case launched – for protection from drifting second-hand smoke)
Sun	Popular tabloid	Su152	09/11/2015	110	Murderer whinges at 'rip-off' cigs in jail	Constructs of prison / prisoners incl tobacco-related culture

Paper	Genre	ID	Date	N words	Article headline	Broad topic
Times	Serious newspaper	Ti118	17/12/2015	153	Killer loses appeal over smoke risk	Legal cases (Gage – case lost)
Herald (Scottish national)	Serious newspaper	HE135	17/12/2015	252	Prisoner loses legal fight over passive smoking	Legal cases (Gage – case lost)
Sun	Popular tabloid	Su153	17/12/2015	91	Cell cigs claim axe	Legal cases (Gage – case lost)
Evening Times (Glasgow, Scotland)	Scottish local	Et164	17/12/2015	146	Anti-smoking prisoner fails in court plea	Legal cases (Gage – case lost)
Guardian	Serious newspaper	Gu002	17/12/2015	351	Victorian police arrest 13 for Ravenhall remand centre riot over smoking ban; Prisoners caused an estimated $10m damage at the maximum security prison in June when ban on smoking in Victorian prisons was introduced	Ravenhall riot / Australian smoke-free prisons
Daily Mirror	Popular tabloid	Mi125	27/12/2015	49	Jail cigs habit dying snout	E-cigarettes in E&W prisons
People	Popular tabloid	Pe042	27/12/2015	71	E-cigs a hit in prisons	E-cigarettes in E&W prisons
Scottish Daily Mail	Middle market tabloid	DM	29/12/2015	165	Jailbirds are bookworms	Constructs of prison / prisoners incl tobacco-related culture
Times	Serious newspaper	Ti116	12/01/2016	482	Prison assault on ex-soldier was 'terrorist attack'	Constructs of prison / prisoners incl tobacco-related culture
Sun	Popular tabloid	Su018	16/02/2016	63	Fags for lags	Legal cases (Black - MoJ challenge launched)
Times	Serious newspaper	Ti029	16/02/2016	110	Government challenge to prison smoking	Legal cases (Black - MoJ challenge launched)
Daily Mirror	Popular tabloid	Mi133	17/02/2016	75	Snag for lags of fag break	Constructs of prison / prisoners incl tobacco-related culture
Guardian	Serious newspaper	Gu010	22/02/2016	4,004	Inside Wandsworth prison: drug drones and demoralised staff; The Guardian has been granted unprecedented access to two prisons to see the impact of funding cuts. In the first of two reports, Amelia Gentleman finds broken windows and bored inmates at the UK's most overcrowded jail	Constructs of prison / prisoners incl tobacco-related culture

Paper	Genre	ID	Date	N words	Article headline	Broad topic
Carrick Gazette (Girvan, Scotland)	Scottish local	CG169	29/02/2016	411	NHS Ayrshire and Arran become the first board to go tobacco free	General re tobacco / e-cigarette smoking (bans), mention of prison(ers)
Daily Mirror	Popular tabloid	Mi128	06/03/2016	119	Non-smoker lags to cash in on fag fear	Prison second-hand smoke levels and risks
People	Popular tabloid	Pe134	06/03/2016	286	Jail Ciggies Compo Fear; Exclusive	Prison second-hand smoke levels and risks
Guardian	Serious newspaper	Gu009	08/03/2016	643	Teenage detainees who climbed on to roof could face damage charges; Six children aged between 15 and 17 allegedly smashed windows, skylights and air-conditioning units at Parkville youth justice centre in Melbourne	Ravenhall riot / Australian smoke-free prisons
Guardian	Serious newspaper	Gu001	09/03/2016	582	Prison smoking ban overturned by court of appeal; Government lawyers had argued that a compulsory ban could cause discipline problems and risk staff and prisoner safety	Legal cases (Black – MoJ challenge won)
Daily Mirror	Popular tabloid	Mi130	09/03/2016	16	Prison ciggie row	Constructs of prison / prisoners incl tobacco-related culture
Times	Serious newspaper	Ti026	09/03/2016	189	Smoking ban does not apply to jails	Legal cases (Black – MoJ challenge won)
Daily Mirror	Popular tabloid	Mi034	09/03/2016	301	Lags free to smoke fags; Workplace cig ban does not apply to jails	Legal cases (Black – MoJ challenge won)
Daily Telegraph	Serious newspaper	DT038	09/03/2016	320	Prisoners can smoke in cells, judges rule	Legal cases (Black – MoJ challenge won)
Daily Mirror	Popular tabloid	Mi127	17/03/2016	91	Inquiry into dissidents' cigar celebration in jail	Constructs of prison / prisoners incl tobacco-related culture
Times	Serious newspaper	Ti025	17/03/2016	66	Romania smoking ban	General re tobacco / e-cigarette smoking (bans), mention of prison(ers)
Sunday Times	Serious newspaper	STi157	20/03/2016	2,010	1916 Ireland's bloody legacy; On the eve of the 100th anniversary of the Easter Rising, the killing of a Belfast prison officer reminds us that for some the revolution is unfinished. The historian Ruth Dudley Edwards shows how the seven leaders of the uprising spawned a civil war and the Troubles 'The only way of starting a war was to kill, so we killed some police'	Constructs of prison / prisoners incl tobacco-related culture
Scotsman (Scottish national)	Serious newspaper	Sc160	06/04/2016	741	How can smoking be a lifestyle choice when most want to stop?	General re tobacco / e-cigarette smoking (bans), mention of prison(ers)

**Paper**	**Genre**	**ID**	**Date**	**N words**	**Article headline**	**Broad topic**
Times	Serious newspaper	Ti028	13/04/2016	268	Inmate death blamed on smoking ban	E&W smoke-free prisons incident
Daily Mirror	Popular tabloid	Mi036	13/04/2016	137	Prisoner 'kills himself over jail ban on cigarettes'	E&W smoke-free prisons incident
Times	Serious newspaper	Ti027	06/05/2016	589	Crown exempt from smoking ban; Law Report	Legal cases<br>(Black – MoJ challenge won)
Sun	Popular tabloid	Su022	10/05/2016	1,174	Brady: 50 years of hatred; Moor murderer shocking evil is revealed by new letters. Now UK's longest serving offender hopes for terrorist attacks in Britain. 78 and frail but sneers at his carers	Constructs of prison / prisoners incl tobacco-related culture
Aberdeen Press and Journal (Aberdeen, Scotland)	Scottish local	AP165	17/05/2016	363	'Skin left peeling off man's face after jail row'	Constructs of prison / prisoners incl tobacco-related culture
