# Supplementary material for: UK news media representations of smoking, smoking policies and tobacco bans in prisons
Source: Tob Control. 2018 Feb 19;27(6):622–30. doi: 10.1136/tobaccocontrol-2017-053868 (PMC6252368; doi:10.1136/tobaccocontrol-2017-053868)
Supplement: Supplementary data [file tobaccocontrol-2017-053868supp004.pdf]

Supplementary 4: Table showing broadcasts – ordered chronologically throughout search period with broadcaster and channel / station, tv / radio, ID, date and time, word count, description and broad topic

Broadcaster and channel / station	TV / radio	ID	Date	N words	Description	Broad topic
BBC News 24	TV	BBC179	05/03/2015 (11:15-12:20)	38	News headlines on UK national 24-hour rolling tv news network	Legal cases (Black - ruling that E&W ban on smoking in public places applies in prisons)
BBC Radio 4	Radio	Rad181	22/07/2015 (06:00)	174	News headlines on UK national speech-based radio station	Anticipation / announcement of E&W smoke-free prisons
BBC 1 Scotland	TV	BSc182	22/07/2015 (06:00)	45	News headlines on the Scottish variation of the UK-wide BBC flagship TV channel	Anticipation / announcement of E&W smoke-free prisons
BBC Radio 4	Radio	Rad167	22/07/2015 (08:00)	96	News headlines on UK national speech-based radio station	Anticipation / announcement of E&W smoke-free prisons
BBC News 24	TV	BBC166 / BBC165	29/09/2015 (16:15)	336	News headlines and subsequent brief background on UK national 24-hour rolling tv news network	Anticipation / announcement of E&W smoke-free prisons
BBC Radio 4	Radio	Rad180a	29/09/2015 (17:00-18:00)	1,041	News headlines and subsequent discussion with members of the Howard League for Penal Reform (UK charity working for less crime, safer communities and fewer people in prison) and the Proclaim Trust (UK evangelical movement doing outreach work with prisoners) on UK national speech-based radio station	Anticipation / announcement of E&W smoke-free prisons
BBC Radio 4	Radio	Rad180b	29/09/2015 (18:00-23:00)	Range 45-80	Hourly news headlines on UK national speech-based radio station	Anticipation / announcement of E&W smoke-free prisons
BBC News 24	TV	BBC178	29/09/2015 (19:20)	487	News headlines and immediate discussion with member of UK Prison Reform Trust (independent UK charity working to create a just, humane and effective penal system) on UK national 24-hour rolling tv news network	Anticipation / announcement of E&W smoke-free prisons
Sky News	TV	SKY171	29/09/2015 (22:24)	345	News headline and summary of several stakeholder comments on UK-based international 24-hour rolling TV news network	Anticipation / announcement of E&W smoke-free prisons
Sky News	TV	SKY170	30/10/2015 (12:04)	515	Several stakeholder comments on 24-hour rolling TV news network	Anticipation / announcement of E&W smoke-free prisons
BBC Radio 5 Live	Radio	BRL183	09/11/2015 (10:25)	2,858	Discussion on UK national radio station that specialises in live news, phone-ins, interviews and sports commentaries	Constructs of prison / prisoners incl tobacco-related culture
BBC News 24	TV	BBC177	18/01/2016 (10:10-10:20)	1,688	Discussion with member of Prison Officers' Association, prisoners and representative of prisoner rehabilitation charity on weekday current affairs programme with remit covering original stories, exclusive interviews and audience debates	Anticipation / announcement of E&W smoke-free prisons

**Broadcaster and channel / station**	**TV / radio**	**ID**	**Date**	**N words**	**Description**	**Broad topic**
BBC News 24	TV	BBC175	08/03/2016 (10:50)	89	News headlines and subsequent brief background on UK national 24-hour rolling tv news network	Legal cases<br>(Black – MoJ challenge won)
BBC News 24	TV	BBC174	08/03/2016 (11:20-11:25)	321	News headlines and subsequent background on UK national 24-hour rolling tv news network	Legal cases<br>(Black – MoJ challenge won)
BBC News 24	TV	BBC173	08/03/2016 (12:25)	75	News headlines on UK national 24-hour rolling tv news network	Legal cases<br>(Black – MoJ challenge won)
BBC News 24	TV	BBC172	08/03/2016 (16:20)	75 (identical to above)	News headlines on UK national 24-hour rolling tv news network	Legal cases<br>(Black – MoJ challenge won)
